# RAN-Binding Protein 9 is Involved in Alternative Splicing and is Critical for Male Germ Cell Development and Male Fertility

**DOI:** 10.1371/journal.pgen.1004825

**Published:** 2014-12-04

**Authors:** Jianqiang Bao, Chong Tang, Jiachen Li, Ying Zhang, Bhupal P. Bhetwal, Huili Zheng, Wei Yan

**Affiliations:** Department of Physiology and Cell Biology, University of Nevada Reno School of Medicine, Reno, Nevada, United States of America; Stanford University School of Medicine, United States of America

## Abstract

As a member of the large Ran-binding protein family, Ran-binding protein 9 (RANBP9) has been suggested to play a critical role in diverse cellular functions in somatic cell lineages *in vitro*, and this is further supported by the neonatal lethality phenotype in *Ranbp9* global knockout mice. However, the exact molecular actions of RANBP9 remain largely unknown. By inactivation of *Ranbp9* specifically in testicular somatic and spermatogenic cells, we discovered that *Ranbp9* was dispensable for Sertoli cell development and functions, but critical for male germ cell development and male fertility. RIP-Seq and proteomic analyses revealed that RANBP9 was associated with multiple key splicing factors and directly targeted >2,300 mRNAs in spermatocytes and round spermatids. Many of the RANBP9 target and non-target mRNAs either displayed aberrant splicing patterns or were dysregulated in the absence of *Ranbp9*. Our data uncovered a novel role of *Ranbp9* in regulating alternative splicing in spermatogenic cells, which is critical for normal spermatogenesis and male fertility.

## Author Summary

Male fertility depends on successful production of functional sperm. Sperm are produced through spermatogenesis, a process of male germ cell proliferation and differentiation in the testis. Most of the genes involved in spermatogenesis are transcribed and processed into multiple isoforms, which are mainly achieved through alternative splicing. The testis-specific transcriptome, characterized by male germ cell-specific alternative splicing patterns, has been shown to be essential for successful spermatogenesis. However, how these male germ cells-specific alternative splicing events are regulated remains largely unknown. Here, we report that RANBP9 is involved in alternative splicing events that are critical for male germ cell development, and dysfunction of RANBP9 leads to disrupted spermatogenesis and compromised male fertility.

## Introduction

Male infertility affects 1 out of 20 men of their reproductive age world-wide and the underlying causes remain largely unknown [1]. Production of functional sperm is achieved through a complex process termed spermatogenesis, which can be divided into three phases, i.e. mitotic, meiotic and haploid. During the mitotic phase, spermatogonia proliferate, differentiate and eventually enter the meiotic phase, in which spermatocytes undergo homologous recombination-mediated crossover followed by two consecutive meiotic cell divisions, and become round spermatids. Haploid round spermatids then undergo a lengthy differentiation process termed spermiogenesis, during which they transform into functionally competent spermatozoa before leaving the seminiferous epithelium for further maturation in the epididymis. Such a complex process requires rigorous spatiotemporal regulation of gene expression at both the transcriptional and post-transcriptional levels. It has long been known that regulation of gene expression depends on the orderly compartmentalization of different regulators within the cells [2]. For example, DNA replication and transcription occur inside the nucleus, while protein translation takes place in the cytoplasm. Thus, transport of macromolecular complexes across the nuclear membrane, termed nucleocytoplasmic transport, occurs frequently through a specialized structure called the nuclear pore complex (NPC) [3]. A large number of soluble transport receptors involved in either nuclear import or export have been identified, and the majority belong to a protein superfamily, members of which display structural homology to importin β (also called karyopherin β), a nuclear import receptor and a key mediator of nuclear localization signal (NLS)-dependent transport [3], [4]. These members can be further categorized into importins or exportins based on their transport directions across the nuclear envelope. For instance, Exportin-5 is responsible for transporting its cargo of hairpin miRNA precursors from the nucleus to the cytoplasm [5].

Numerous cofactors have been found to bind importins or exportins to facilitate nucleocytoplasmic transport, e.g. Ran-binding protein family (RanBP). Ran-binding protein 1 (RANBP1) binds the GTP-bound form of RAN and stimulates the rate of GTP hydrolysis induced by the RANGAP [6], [7]. Ran binding protein 3 (RANBP3) can facilitate the transport of CRM (Exportin-1)-mediated mRNA precursors and nuclear export signal (NES)-containing proteins in eukaryotes [8]. Ran binding protein 5 (RANBP5) represents a novel transport factor because it binds the NPC with a substrate specificity distinct from importin-α/β member receptors [9]. RANBP9, also called RANBPM, is a 90 kD protein containing five conserved functional domains, including the N-terminal proline-rich domain (PRD), a SPRY domain, a lissencephaly type-I-like homology (LisH) motif, a C-terminal to LisH (CTLH) motif, and a C-terminal CRA motif [10], [11]. Increasing lines of evidence suggest that these conserved domains are responsible for mediating interactions of RANBP9 with >45 other protein partners in various somatic cell types under different physiological conditions [12]–[21].

In germ cells, RANBP9 has been shown to interact with DDX4 (also called MVH for mouse Vasa homolog), a germline-specific RNA helicase [22], and also with GASZ, a germ cell protein abundantly expressed in spermatocytes and essential for transposon suppression [23]. Global *Ranbp9* knockout (KO) mice generated using the gene-trapped strategy exhibit severely impaired spermatogenesis and premature ovarian failure [24], [25]. However, further analyses were hindered due to the neonatal lethality phenotype [24], [25]. To dissect the cell-specific biological importance of *Ranbp9* and its molecular actions, we generated germ cell- and Sertoli cell-specific *Ranbp9* conditional knockout (cKO) mouse lines. By studying these *Ranbp9* cKO mice, we discovered that RANBP9 interacts with numerous key splicing factors, and is involved in alternative splicing of numerous mRNAs during the meiotic and haploid phases of spermatogenesis.

## Results

### RANBP9 is preferentially expressed in the testis and is mainly localized to the nuclei of spermatocytes and spermatids

**Figure 1:**
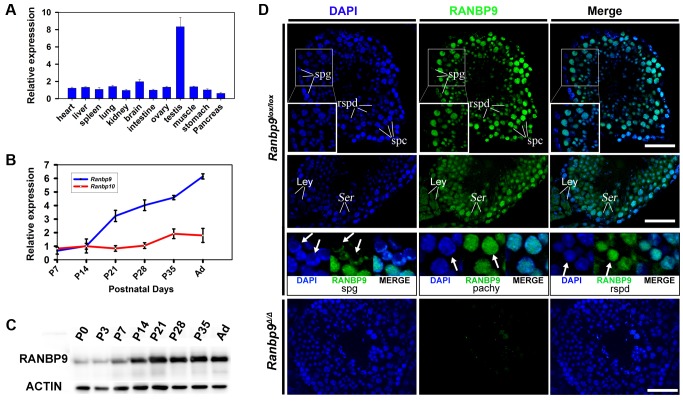
Expression profiles of *Ranbp9* during testicular development and spermatogenesis in mice. (A) qPCR analyses of *Ranbp9* mRNA levels in multiple organs in mice. Data are presented as mean ± SEM, n = 3. (B) Expression of *Ranbp9* and *Ranbp10* during postnatal testicular development. Levels of *Ranbp9* and *Ranbp10* mRNAs in developing testes at postnatal day 7 (P7), P14, P21, P28, P35, and in adult (Ad) were analyzed using qPCR. Data are presented as mean ± SEM, n = 3. (C) Expression of RANBP9 protein during postnatal testicular development. Levels of RANBP9 in the testes from newborn (P0), postnatal day 3 (P3), P7, P14, P21, P28, and P35 and adult male mice were determined using western blot analyses. ACTIN was used as a loading control. (D) Immunofluorescent detection of RANBP9 in homozygous *Ranbp9* flox (*Ranbp9^lox/lox^*) and *Ranbp9* global knockout (*Ranbp9^Δ/Δ^*) testes. In *Ranbp9^lox/lox^* testes, RANBP9 immunoreactivity was mostly detected in the nucleus of spermatocytes (spc) and spermatids (spd). Insets show the digitally magnified view of the framed area. RANBP9 was also detected in the nucleolus of Sertoli cells (Ser), and in both the cytoplasm and the nucleus in interstitial Leydig cells (Ley) (Middle panels). While the nucleus was partially RANBP9-positive in a subpopulation of spermatogonia (spg), RANBP9 staining covered the entire nucleus in both pachytene spermatocytes (pachy) and round spermatids (rspd) (Lower panels). In *Ranbp9^Δ/Δ^* testes, RANBP9 staining was completely absent. Scale bar = 50 µm.

qPCR analyses detected *Ranbp9* mRNA expression in all 12 organs examined, with the highest levels in the testis and the second highest in brain (Figure 1A). This expression profile is consistent with that reported previously [11], [18], [26]. During postnatal testicular development, levels of *Ranbp9* mRNA increased drastically from postnatal day 14 (P14) onward (Figure 1B), coinciding with the first appearance of pachytene spermatocytes in the seminiferous epithelium. Among all ten members of the RAN-binding protein family, RANBP9 shares the highest homology with RANBP10 in both amino acid sequences and conserved domains. The only difference lies in the N-terminal proline and glutamine-rich region, which is present in RANBP9, but absent in RANBP10 [11], [27]–[29]. Therefore, we also analyzed the expression profile of *Ranbp10*. Interestingly, while levels of *Ranbp9* mRNA kept increasing after P14 and peaked in adulthood, *Ranbp10* mRNA levels remained roughly constant during postnatal testicular development (Figure 1B). The differential expression patterns suggest that these two Ran-binding proteins are differentially regulated in different cell types, and thus, may have non-redundant roles during testicular development and spermatogenesis.

Using a well-characterized monoclonal *Ranbp9* antibody [30], we examined RANBP9 protein expression profiles during testicular development using Western blot analyses (Figure 1C). Similar to the *Ranbp9* mRNA expression profile (Figure 1B), levels of RANBP9 increased significantly at P14 and remained at elevated levels afterwards, suggesting that RANBP9 is abundantly expressed in pachytene spermatocytes and spermatids, as these two cell types constitute the major cell types within the testes after P14. To further define its subcellular localization, we performed immunofluorescence staining to detect RANBP9 using testicular cryosections. RANBP9 protein was predominantly localized to the nucleus of spermatocytes at all stages (i.e., preleptotene, leptotene, zygotene, pachytene and diplotene), and to the nucleus of spermatids of steps 1–15 (Figure 1D). RANBP9 levels were higher in pachytene spermatocytes and round spermatids (steps 1–8), and progressively decreased in elongated spermatids (from steps 9 to 15) (Figure 1D). Weak RANBP9 staining was also detected in the nucleoli of Sertoli cells, and in both the nucleus and the cytoplasm in interstitial Leydig cells (Figure 1D). Interestingly, immunoreactivity was detected in only a portion of the nucleus in RANBP9-positive spermatogonia, whereas the entire nucleus was largely homogeneously stained in pachytene spermatocytes and spermatids (Figure 1D). As a negative control, specific RANBP9 signals were completely absent in global *Ranbp9* knockout (*Ranbp9^Δ/Δ^*) testes. Taken together, RANBP9 is predominantly localized to the nuclei of spermatocytes and spermatids in the adult murine testes.

### Generation of germ cell- and Sertoli cell-specific *Ranbp9* conditional knockout mice

**Figure 2:**
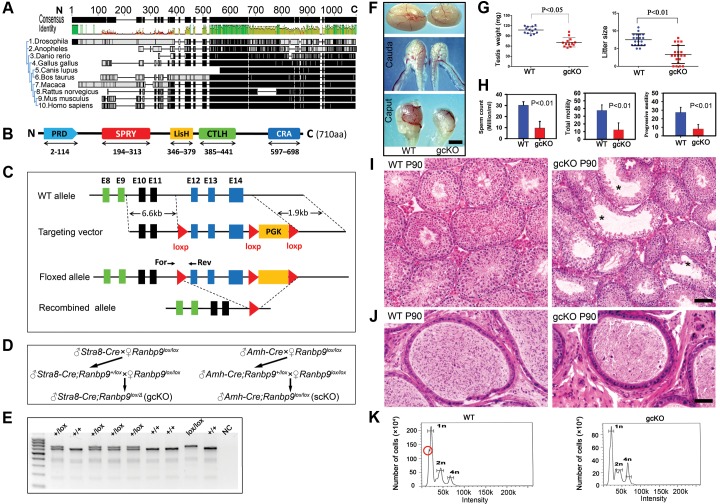
Conditional inactivation of *Ranbp9* reveals that male germ cell *Ranbp9* is required for normal spermatogenesis and male fertility. (A) A high degree of conservation of RANBP9 in amino acid sequences, especially at the C-terminus, among ten eukaryotic species. (B) Schematic illustration of the five conserved domains in murine RANBP9, including a proline-rich domain (PRD) at the N-terminus, SPRY, LiSH and CTLH domains in the middle, and a CRA domain at the C-terminus. (C) Schematic representation of the targeting strategy for generating a floxed *Ranbp9* allele (*Ranbp9^lox^*) through homologous recombination in the murine embryonic stem cells. Exons 12∼14 encode the CRA domain and will be deleted after Cre-mediated recombination. E stands for Exon. Positions of the forward (For) and reverse (Rev) primers used for genotyping are shown. (D) Breeding schemes used for generating germ cell-specific (gcKO) and Sertoli cell-specific (scKO) *Ranbp9* conditional knockout mice. (E) Representative PCR genotyping results showing that the floxed (lox) and the WT (+) alleles can be detected as a larger (653 bp) and a shorter (605 bp) bands, respectively. (F) Gross morphology of the testis and the epididymis from WT and gcKO mice at the age of 12 weeks. Scale bar = 1 mm. (G) Testis weight and litter size of 12-week-old gcKO and WT male mice. The gcKO males display significantly reduced testis weight (∼65% of WT) and smaller litter size (∼half of WT) (p<0.05, n = 13). (H) Sperm counts, and total and progressive sperm motility of 12-week-old gcKO and WT male mice, as determined by CASA. Adult gcKO male mice exhibit significantly reduced sperm concentration, total and progressive motility, as compared to age-matched WT mice. Data are presented as mean ± SEM, n = 3. (I) Testicular histology of WT and gcKO mice at postnatal day 90 (P90). Large vacuoles (marked with *) indicative of active depletion of spermatocytes and/or spermatids through sloughing are often seen in seminiferous tubules of the gcKO testes. Scale bar = 50 µm. (J) Epididymal histology of WT and gcKO mice at postnatal day 90 (P90). The WT cauda epididymis is filled with fully developed spermatozoa, whereas the gcKO cauda epididymis contains numerous degenerating/degenerated spermatids or spermatocytes. Scale bar = 60 µm. (K) Flow cytometry-based cell counting analyses showing the altered proportions of three major germ cell types, including haploid spermatids (1n), spermatogonia and somatic cells (2n), and spermatocytes (4n) in WT and gcKO testes. Red circle denotes the fraction representing elongated spermatids and spermatozoa, which is mostly absent in the gcKO testes.

Global ablation of *Ranbp9* leads to neonatal lethality [24], [25], precluding further investigation of the roles of *Ranbp9* in postnatal development and during adulthood. To overcome this obstacle, we generated a *Ranbp9* loxP mouse line, which allowed us to inactivate *Ranbp9* in a cell- or tissue-specific manner using the Cre-loxP system. Full-length RANBP9 protein contains five consensus domains [11]–[20], while only the C-terminus is highly conserved across multiple eukaryotic species (Figure 2A,B, Table S1), implying that the C-terminus could be essential for proper RANBP9 functions *in vivo*. We, therefore, decided to generate a *Ranbp9* floxed allele (*Ranbp9^lox^*) by inserting two loxP cassettes, one before exon 12 and the other after exon 14 (Figure 2C). In this way, the C-terminal CRA domain of RANBP9 would be removed in the progeny after Cre-mediated recombination in the targeted cell types (Figure 2C). The *Ranbp9^lox/lox^* mice are viable and healthy, suggesting the *Ranbp9* flox allele that we created is fully functional. To inactivate *Ranbp9* exclusively in Sertoli cells, an *Amh-Cre* deletor line with Cre expression in Sertoli cells at ∼E12.5 [31], was crossed with *Ranbp9^lox/lox^* mice to generate Sertoli cell-specific *Ranbp9* conditional knockout (*Amh-Cre; Ranbp9^lox/lox^*, hereafter named scKO) mice (Figure 2D). Similarly, by crossing *Ranbp9^lox/lox^* mice with *Stra8-Cre* mice [32], we generated postnatal male gcKO (*Stra8-Cre;Ranbp9^lox/Δ^*) mice (Figure 2D), in which *Ranbp9* is inactivated exclusively in developing male germ cells starting at P3 [33]. PCR genotyping (Figure 2E) was used to distinguish WT and loxP alleles, and immunofluorescence staining (Figure 1D) confirmed that the recombined *Ranbp9* allele (*Ranbp9^Δ^*) was truly null. Thus, we successfully generated *Ranbp9* gcKO and scKO mice.

### *Ranbp9* is dispensable for Sertoli cell development, but is critical for male germ cell development and male fertility

scKO male mice developed normally and their fertility was comparable to that of WT males. Further examination revealed normal testicular histology, sperm counts and motility (Figure S1), suggesting that *Ranbp9* is dispensable for normal Sertoli cell development and function. In contrast, despite normal body size and weight, adult gcKO male mice at the age of 12 weeks displayed reduced testis size and decreased testis weight compared to age-matched WT male mice [71.1 mg±3.8 (gcKO) vs. 107.5 mg±2.8 (WT), n = 13, p<0.05] (Figure 2F, G). Caput and cauda epididymides of the gcKO male mice were also smaller compared to those of their age-matched WT males (Figure 2F). Computer-assisted semen analyses (CASA) of cauda epididymal sperm revealed that sperm counts, total motility and progressive motility of the gcKO sperm were all lower than those of age-matched WT sperm (Figure 2H). Microscopic examination of epididymal sperm revealed a variety of morphological abnormalities in both the sperm head (e.g. crooked, round, or bent) and the flagellum (e.g. coiled or headless) (Figure S2).

During the first wave of spermatogenesis, no differences in testicular histology were observed between the WT and gcKO testes (Figure S3). However, in 3-month old gcKO testes, the seminiferous epithelium appeared to be much thinner compared to WT testes, and contained numerous vacuoles, indicative of active germ cell depletion (Figure 2I, Figure S3), which was further confirmed by the presence of numerous degenerating/degenerated spermatids, or even spermatocytes in the epididymis of the gcKO males (Figure 2J). Flow cytometry-based cell counting unveiled a proportional increase of meiotic cells (i.e., spermatocytes) (4n) (23% in gcKO *vs.* 11% in WT), and a proportional decrease in haploid cells (i.e., spermatids) (1n) (61% in gcKO *vs.* 70% in WT), despite lowered total cell number in both populations in gcKO testes compared to age-matched WT males (Figure 2K). These results indicate active depletion of spermatocytes and spermatids in the gcKO testes, which is consistent with the histological analyses described above. The gcKO males exhibited markedly reduced fertility compared to WT controls in 5-month-long fertility tests (Litter size: 3.4±0.6 vs. 7.5±0.4, n = 20, p<0.01) (Figure 2G) due to spermatogenic disruptions. Notably, some gcKO adult males were completely infertile, whereas others displayed close-to-normal fertility (Figure 2G). It is unlikely that this phenotypic heterogeneity resulted from the partial penetrance of Stra8-Cre, as we reported previously [33], because we only used *Stra8-Cre;Ranbp9^+/lox^* males, whose germ cells have a genotype of *Ranbp9^+/Δ^*, to cross with *Ranbp9^lox/lox^* females in our breeding schemes (Figure 2D). Nevertheless, fertility of the majority of the gcKO males (∼75%, 15 out of 20) was significantly reduced (Figure 2G). Taken together, our data demonstrate that *Ranbp9* has a critical role in spermatogenesis, but is dispensable for normal Sertoli cell development and function.

### RANBP9-dependent extrinsic factors also contribute to successful spermatogenesis and male fertility

As described above, *Ranbp9* mRNA is ubiquitously expressed in multiple organs (Figure 1A), and within the testis, RANBP9 is expressed in both spermatogenic and somatic cells (e.g. Sertoli cells and Leydig cells) (Figure 1D). Non-germ cell functions of RANBP9 can be revealed by phenotypic differences between global *Ranbp9* KO and gcKO mice. Therefore, we generated mice homozygous for the Cre-mediated deletion alleles (*Ranbp9* ^Δ/Δ^), which allowed us to not only evaluate the effects of global inactivation of *Ranbp9* on overall development and fertility, but also compare our data with those from two previous reports in which *Ranbp9* expression was globally blocked using a gene-trap strategy [24], [25]. Similar to the mice homozygous for the gene-trapped *Ranbp9* alleles [24], [25], *Ranbp9^Δ/Δ^* mice also exhibited postnatal growth retardation (Figure S4A, C and F), and neonatal lethality probably due to developmental defects in brain [25]. Among all the *Ranbp9^Δ/Δ^* pups born, only ∼6% survived to adulthood. The weights of whole body or the testis of *Ranbp9* ^Δ/Δ^ mice were significantly lower compared to those of age-matched WT littermates although there appeared to be no difference in the testis/body weight index (ratio of testis weight/body weight) (Figure S4B, C, D, E, F). Histological analyses revealed that spermatogenesis in *Ranbp9^Δ/Δ^* testes proceeded through meiosis and reached round spermatid and even elongated spermatid stages, despite drastically reduced total number of spermatogenic cells (Figure S4G). This is different from the meiotic arrest phenotype in the male mice homozygous for the gene-trapped *Ranbp9* allele [24]. Nevertheless, *Ranbp9*-null spermatocytes and spermatids appeared to be constantly depleted (Figure S4G), leading to a complete lack of spermatozoa in the epididymis, resembling azoospermia in humans (Figure S4H). These *Ranbp9*-null male mice, even if they survived, were completely infertile. In general, the developmental defects, e.g. growth retardation and neonatal lethality, suggest that *Ranbp9* has a critical role in the development of other vital organs, the most likely being the brain [25].

### Enhanced spermatocyte apoptosis and spermatid depletion in *Ranbp9* gcKO testes result from unrepaired DNA double-strand breaks and aberrant expression of key spermiogenic genes

**Figure 3:**
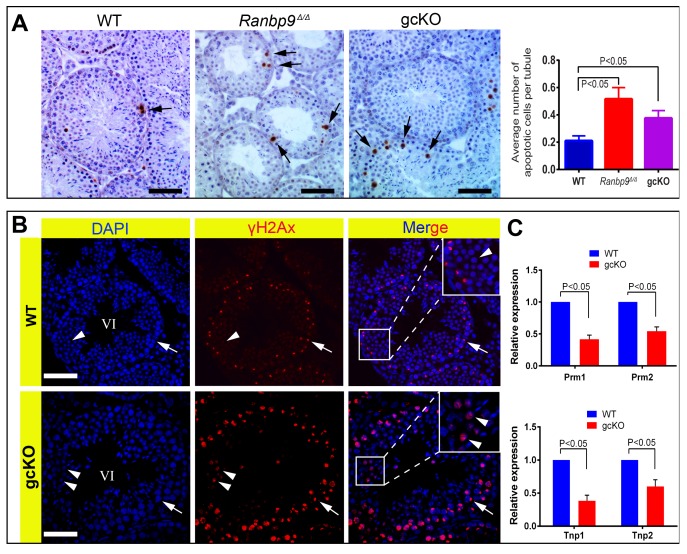
*Ranbp9* deficiency causes male germ cell apoptosis and DNA double-strand breaks. (A) TUNEL assays on WT, *Ranbp9* global KO (*Ranbp9^Δ/Δ^*) and gcKO testes. Arrows point to apoptotic cells stained in brown. Scale bar = 50 µm. Significantly increased average number of apoptotic cells is observed in both *Ranbp9* ^Δ/Δ^ and gcKO testis (the far right panel). >60 cross-sections were scored for the average number of apoptotic cells per tubule for each genotype. Three mice of each genotype were analyzed, and data were presented as mean ± SD, n = 3. (B) Immunofluorescence staining of γH2AX in seminiferous tubules of WT and gcKO testes at ∼stage VI. In WT seminiferous tubules, γH2AX immunoreactivity is mostly confined to the XY body (arrows) in pachytene spermatocytes and completely absent in round spermatids (arrowheads). In contrast, in gcKO seminiferous tubules, numerous round spermatids exhibit strong γH2AX staining (arrowheads) in addition to its normal localization in the XY body (arrow) in pachytene spermatocytes. (C) qPCR analyses showing significantly reduced levels of *Prm1*, *Prm2*, *Tnp1* and *Tnp2* mRNAs in 6-week old *Ranbp9* gcKO testes. Data are presented as mean ± SEM, n = 3.

As described above, enhanced depletion of spermatocytes and spermatids, and the production of deformed spermatozoa were only observed and appeared to be the main phenotype in gcKO male mice older than 2 months. To explore the underlying mechanism, we examined apoptosis and DNA double-strand breaks (DSBs) using TUNEL and γH2AX (a protein marker for DSBs) immunohistochemistry, respectively. As expected, a ∼2-fold increase in apoptotic cells, which were mainly spermatocytes and early round spermatids, was detected in both gcKO and *Ranbp9^Δ/Δ^* testes, as compared to WT controls (Figure 3A). During normal spermatogenesis, γH2AX is localized to the nuclei of leptotene and zygotene spermatocytes, and the XY body (i.e. sex body) of pachytene spermatocytes, as well as the nuclei of elongating spermatids because of active DSBs under physiological conditions [34]–[36]. However, in *Ranbp9* gcKO seminiferous tubules at ∼stage VI, while γH2AX staining was typically confined to the XY body of pachytene spermatocytes, ∼30–45% of round spermatids displayed strong γH2AX staining (Figure 3B, lower panels), suggesting DNA DSBs. In contrast, γH2AX staining was almost completely absent in WT round spermatids in tubules of roughly the same stage (Figure 3B, upper panels). We also analyzed levels of *Tnp1*, *Tnp2*, *Prm1* and *Prm2* because these genes are essential for proper chromatin condensation and aberrant expression of these genes leads to persistent DSBs and deformed spermatozoa with head abnormalities [37]–[40]. Indeed, qPCR analyses revealed markedly reduced expression levels of all four mRNAs (Figure 3C), suggesting that the normal spermiogenic transcriptome requires functional *Ranbp9*, although the defects may well be secondary to the *Ranbp9* ablation.

### RANBP9 is not involved in piRNA biogenesis and transposon silencing

Previous studies have demonstrated that RANBP9 interacts with two germline-specific proteins, DDX4 (also called MVH) and GASZ, both of which are involved in piRNA-mediated transposon silencing in murine testes [23], [41]. We, therefore, examined LINE1 retrotransposon expression at both protein and mRNA levels. As a positive control, LINE1 retrotransposon-derived ORF1 protein was highly expressed in *Miwi2*-null germ cells (Figure S5A). However, ORF1 was undetectable in *Ranbp9* gcKO testis (Figure S5A). mRNA levels of either murine DNA transposons (Charlier, Mariner, MusD, Sine B2, Sine B1) or retrotransposons (LINE1 and IAP) were not up-regulated in either *Ranbp9* gcKO or global KO (*Ranbp9^Δ/Δ^*) testes (Figure S5B, C), suggesting RANBP9, unlike GASZ and DDX4, is not involved in piRNA-mediated transposon silencing in the male germline. The biogenesis of pachytene piRNAs is believed to be an endonuclease-dependent process involving multiple maturation steps, for which the protein effectors remain yet to be identified [42]. The nucleocytoplasmic transport capability of the RAN-binding domain protein family prompted us to explore whether RANBP9 could function as an exportin responsible for exporting piRNA precursors out of the nucleus during pachytene piRNA biogenesis in spermatocytes and spermatids. However, no accumulation of the precursors of four representative piRNAs was observed in *Ranbp9* gcKO testes (Figure S5D). Moreover, we performed small noncoding RNA deep sequencing (sncRNA-Seq), and found no major changes in piRNA transcriptome. Together, these data demonstrate that RANBP9 is not involved in the piRNA pathway.

### RANBP9 participates in alternative splicing by interacting with poly(A) binding proteins (PABPs) and splicing factors in the testis

**Figure 4:**
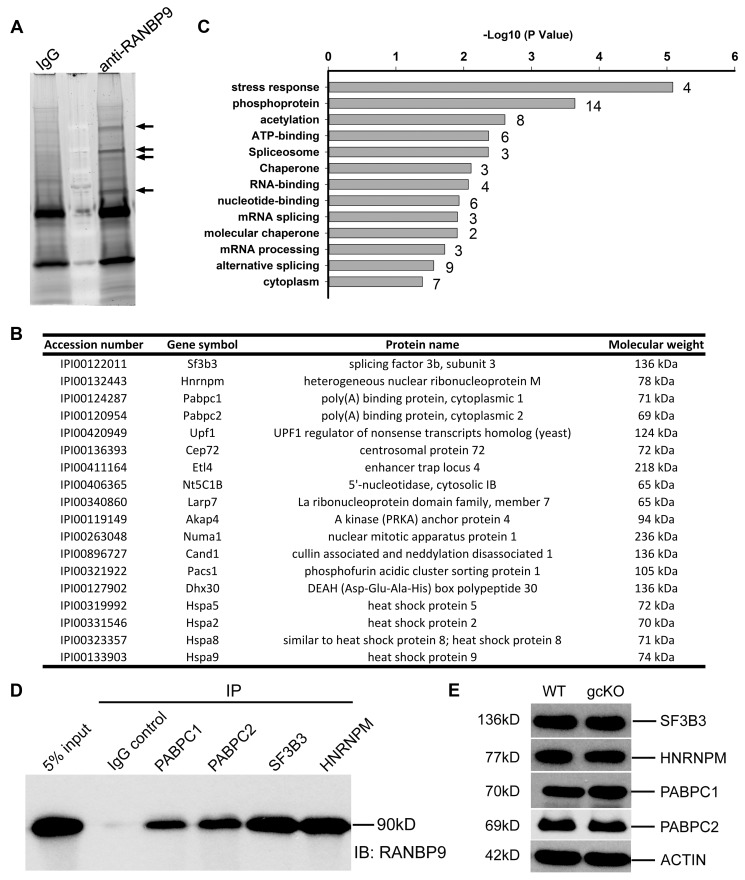
Identification of RANBP9-interacting partners in murine testes using immunoprecipitation followed by mass spectrometry (IP-MS). (A) A representative gel image showing bands representing proteins immunoprecipitated by the monoclonal anti-RANBP9 antibody used or IgG (control). Arrows indicate protein bands unique to the IP products of anti-RANBP9 antibody, which were excised for subsequent MS analyses. (B) A list of 18 RANBP9-interacting partners in murine testes identified by IP-MS. All proteins were detected multiple times in all three biological replicates. (C) Results of gene ontology (GO) term enrichment analyses of RANBP9-interacting proteins. (D) Validation of interactions between RANBP9 and four putative RANBP9-interacting proteins (PABPC1, PABPC2, SF3B3 and HNRNPM) in murine testes by *in vivo* co-immunoprecipitation assays, in which antibodies specific for the four proteins were used for immunoprecipitation (IP) followed by Western blot analyses using a mouse monoclonal anti-RANBP9 antibody. IgG was used as a negative control. (E) A representative Western blot analyses showing levels of four RANBP9-interacting proteins (SF3B3, HNRNPM, PABPC1 and PABPC2) in 6-week old WT and *Ranbp9* gcKO testes.

RANBP9 contains multiple conserved domains, some of which have been shown to mediate interactions between RANBP9 and its numerous partners in various somatic cell types [12]–[15], [30], [43]. The finding that RANBP9 does not play a role in transposon silencing prompted us to define the RANBP9 interactome in the testis. To achieve this goal, we performed immunoprecipitation-mass spectrometry (IP-MS) assays using a well-validated mouse monoclonal RANBP9 antibody [30], [44]. Specific bands identified exclusively in the RANBP9 antibody pull-down products were excised for protein identification using MS (Figure 4A). A total of 18 proteins were repeatedly identified in all three biological replicates (Figure 4B). GO enrichment analyses revealed that 9 out of the18 proteins identified function in RNA-binding/processing biological processes (Figure 4B, C), most notably, alternative splicing. Using *in vivo* co-immunoprecipitation assays, we further validated and confirmed that two poly(A) binding proteins (PABPC1 and PABPC2) and two key splicing factors (SF3B3 and HNRNPM) identified through IP-MS were indeed *bona fide* interacting partners of RANBP9 in the testis (Figure 4D). Importantly, expression levels of all four RANBP9-interacting proteins (SF3B3, HNRNPM PABPC1 and PABPC2) were not significantly affected in gcKO testes, as compared to WT testes (Figure 4E), suggesting that the absence of RANBP9 in spermatogenic cells does not affect the levels of its interacting partners.

**Figure 5:**
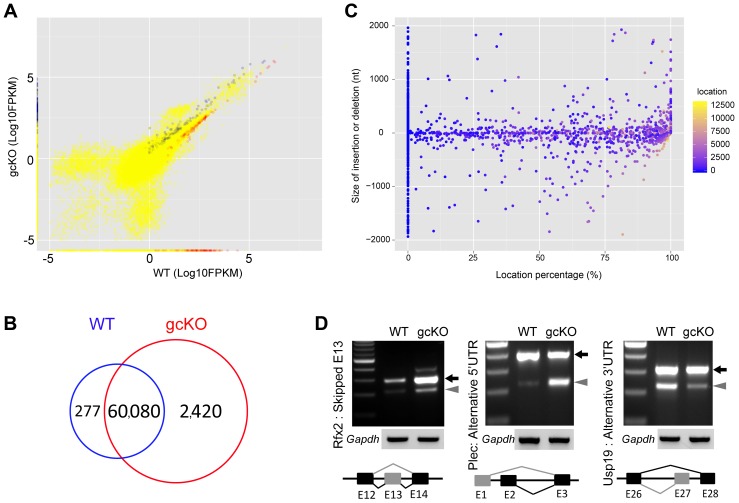
Disruptions of the mRNA transcriptome and alternative splicing patterns in *Ranbp9* gcKO testes. (A) Scatter plot showing significantly de-regulated transcripts in *Ranbp9* gcKO testes compared to WT controls. Blue dots (2,313) represent significantly upregulated transcripts, while red dots (316) denote significantly downregulated transcripts (p<0.05, fold change>2). Yellow dots illustrate unchanged transcripts. (B) Venn diagram showing the number of unique transcript isoforms detected in *Ranbp9* gcKO (2,420) and WT (277) testes. (C) Distribution of 1,816 aberrant splicing events (insertions or deletions) along the entire length of mRNAs in gcKO testes. The y-axis represents the size of insertions (positive values) or deletions (negative values), whereas the x-axis denotes location percentage (splicing location/total transcript size), reflecting the relative position of splicing events along the entire length of the transcripts, e.g., 0% refers to the very 3′end, 50% means the middle of the transcript and 100% indicates the very 5′end. (D) Semi-qPCR-based detection of aberrant alterative splicing patterns in three RANBP9 direct target mRNAs (*Rfx2*, *Plec* and *Usp19*). Lower panels represent the schematic diagram of alternatively spliced exons detected by RNA-Seq analysis. *Gapdh* was used as a loading control.

Given that RANBP9 is mainly confined to the nucleus, and it interacts with key splicing factors, we reasoned that RANBP9 is likely involved in alternative splicing in spermatocytes and spermatids. To explore this possibility, we further performed RNA-Seq analyses using WT and gcKO testes at the age of 6 weeks. We chose this timepoint because germ cell depletion was minimal and thus, the cellular compositions were comparable between gcKO and WT testes (Figure S3). RNA-Seq analyses identified 2,313 upregulated and 316 downregulated genes in gcKO testes as compared to WT controls (p<0.05, fold change >2) (Figure 5A) (Table S2). Further bioinformatic analyses revealed that a total of 2,420 unique transcript isoforms were detected exclusively in the gcKO testes, as compared to only 277 unique transcript isoforms in the WT testes (p<0.1, one way t-test) (Figure 5B). The drastic increase in unique transcript isoforms in gcKO testes suggests that numerous novel isoforms are synthesized in the absence of RANBP9 in spermatogenic cells. To determine whether gcKO unique transcript isoforms represented products of aberrant splicing, we analyzed splicing events using our in-house pipeline, which compares the standard isoforms (defined as those with the highest expression levels in WT testes) with those gcKO-specific/unique isoforms that are homologous to the standard forms (partially matching), so that the differential portions and their locations can be determined. Interestingly, we detected 1,816 aberrant splicing events that occurred in 1,562 unique transcript isoforms (corresponding to 695 genes) in gcKO testes (Tables S3 and S4). By plotting the two types of splicing events (insertions vs. deletions) against the relative position along the entire lengths of gcKO unique transcripts (the 3′UTR, the gene body and the 5′UTR), we found that insertions were detected in the 3′UTR, the gene body and the 5′UTR, whereas deletions were observed in the 5′UTR and the gene body, but not in the 3′UTR (Figure 5C, Tables S3 and S4). These data strongly suggest that RANBP9 plays a critical role in regulating global alternative splicing in adult mouse testes.

### RANBP9 targets >2,300 mRNAs and affects their expression levels at least partially through regulating alternative splicing

**Figure 6:**
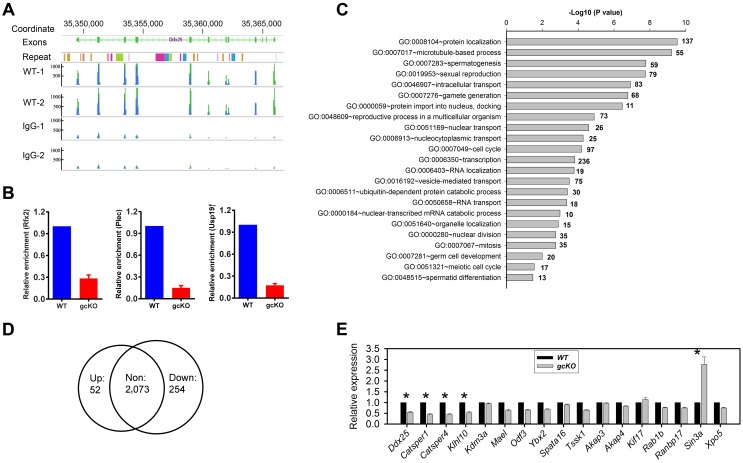
RANBP9 binds numerous mRNAs and affects their expression levels at least partially through affecting alternative splicing. (A) A representative mRNA assembly output showing RIP-Seq reads for *Ddx25* identified from the RIP products using the RANBP9 antibody and IgG (control). (B) qPCR analyses of levels of three RANBP9-bound mRNAs (*Rfx2*, *Plec* and *Usp19*) in WT and gcKO testes. All three are highly enriched in WT compared to gcKO testes, demonstrating the specificity of the anti-RANBP9 antibody used in RIP-Seq assays. (C) GO enrichment analyses of RANBP9-bound mRNAs identified using RIP-Seq. (D) Venn diagram showing the number of up- and down-regulated RANBP9-bound target transcripts in gcKO testes (P<0.05, fold change>1.5). (E) qPCR analyses of levels of 17 RANBP9 target transcripts in 6-week-old WT and gcKO testes. Data are presented as mean ± SEM, and significantly altered levels were marked with * (n = 4, P<0.05).

The conserved domains of RANBP9, including SPRY, LiSH, CTLH and CRA, have been shown to serve as scaffolding modules, mediating interactions between RANBP9 and its protein partners in various types of somatic cells [26], [44]–[46]. Given the potential role of RANBP9 in alternative splicing, it is possible that RANBP9 can directly, or indirectly, bind its target mRNAs through either its own conserved domains or its interacting partners. To identify RANBP9 target mRNAs, we performed RNA-immunoprecipitation followed by next-gen sequencing (RIP-Seq) assays, using a validated RANBP9 monoclonal antibody for genome-wide identification of RANBP9 target transcripts. Annotation of the RIP-Seq data identified a total of 2,379 transcripts that were significantly enriched in the RANBP9 immunoprecipitants (cutoff: P<0.05, fold change>2) (Table S5). For example, *Ddx25*, which encodes a germline granule-specific RNA helicase, was barely detected in control samples in which IgG was used, but was abundantly enriched when the RANBP9 monoclonal antibody was used for the immunoprecipitation of RANBP9 from WT testis lysates (Figure 6A). Using qPCR, we further selected three RANBP9-bound target mRNAs and confirmed that these indeed were predominantly present in RANBP9 immunoprecipitants from WT testes instead of *Ranbp9* gcKO testes (Figure 6B). Interestingly, GO enrichment analyses revealed that RANBP9-bound mRNAs were mostly involved in protein/RNA transport and spermatogenesis (Figure 6C). Given the predominant expression of RANBP9 in the testis, it is not surprising to see that mRNAs targeted by RANBP9 are mostly related to spermatogenesis, especially spermiogenesis. However, the enrichment of proteins involved in protein/RNA transport among all RANBP9-interacting partners is noteworthy because it suggests a potential role of RANBP9 in protein and/or RNA trafficking, which may be related to alternative splicing, or other events.

By comparing RNA-Seq and RIP-Seq results, we found that 154 out of the 695 genes (∼22%) uniquely expressed in gcKO testes were among the 2,379 RANBP9-bound targets. Moreover, among all 2,379 RANBP9-bound targets, 52 transcripts were upregulated, while 254 targets were down regulated in gcKO testes (cutoff: P<0.05, fold change>1.5) (Figure 6D) (Table S6). We chose three RANBP9 target transcripts identified through RIP-Seq and examined their alternative splicing patterns. Consistently, we detected aberrant splicing patterns in *Rfx2*, *Plec* and *Usp19* in gcKO testes (Figure 5D). We further examined levels of 17 RANBP9-bound mRNAs selected based on their known essential functions in spermatogenesis, especially in spermiogenesis [1], [47]. Consistent with the RNA-Seq results, levels of 4 male germ cell-specific mRNAs (*Ddx25*, *Catsper1*, *Catsper4* and *Klhl10*) [48]–[51] were significantly downregulated, whereas levels of *Sin3a* were drastically upregulated (Figure 6E). Taken together, our data suggest that RANBP9 is involved in the alternative splicing of many of its target transcripts that are expressed in spermatocytes and spermatids.

## Discussion

The conditional knockout approach using the Cre-loxP system remains an ideal way to define cell-specific functions of genes, especially those essential for embryonic and perinatal development. The neonatal lethality phenotype of *Ranbp9* global KO mice [24] precludes further analyses of *Ranbp9* functions in postnatal development and adulthood. It is, therefore, essential to generate cell-specific *Ranbp9* conditional knockout mice to delineate the cell-specific function of *Ranbp9*. The absence of any discernable phenotype in scKO mice indicates that *Ranbp9* is dispensable for Sertoli cell development and functions. In contrast, the progressive spermatogenic disruptions and male subfertility or infertility in adult gcKO mice demonstrate the importance of *Ranbp9* in male germ cell development. Interestingly, the testicular phenotype appears to be different between *Ranbp*9 global KO and gcKO mice. Two *Ranbp9* global KO lines, one generated by the gene trap strategy [24] and the other, as reported here, obtained through Cre-loxP-mediated gene deletion, are both completely infertile, whereas the majority of gcKO males are subfertile. This discrepancy hints that extrinsic factors, e.g. endocrine signals from the hypothalamus and the pituitary, or paracrine factors from Sertoli and/or Leydig cells, may also contribute to the spermatogenic disruptions observed in *Ranbp9* global KO males. Thus, RANBP9-dependent functions in somatic cell types may have a role in successful spermatogenesis and male fertility.

To explore the true physiological roles of RANBP9, we adopted the unbiased approaches, including RNA-Seq, IP-MS and RIP-Seq, to identify RANBP9-interacting partners and its potential mRNA targets. >2,400 mRNA transcripts with unique alternative splicing patterns in the gcKO testes suggest that RANBP9 may be involved in the control of alternative splicing. This is further supported by our *in vivo* IP-MS assays, which reveal that RANBP9 interacts with key splicing factors (e.g. SF3B3 and HNRNPM) and poly (A) binding proteins (PABPC1 and PABPC2). PABPC1 and PABPC2 have recently been demonstrated to shuttle between the cytoplasm and the nucleus, and these two PABP proteins participate in pre-mRNA processing in the nucleus, and mRNA metabolism in the cytoplasm [52], [53]. Therefore, it is highly likely that RANBP9 is involved in the nuclear functions of PABPC1 and PABPC2, i.e., pre-mRNA processing (e.g., alternative splicing). The 2,379 transcripts identified in our genome-wide RIP-Seq analyses most likely represent RANBP9 targets. The fact that 154 of the RANBP9 target mRNAs display aberrant splicing patterns in gcKO testes further supports a role of RANBP9 in alternative splicing.

RANBP9 is a member of the large importin/exportin family, which prompted us to first postulate that RANBP9 acts as a nucleocytoplasmic transporter. Given that RANBP9 interacts with the piRNA pathway components, e.g., DDX4/MVH and GASZ [22], [23], we hypothesized that RANBP9 might be responsible for exporting precursor piRNAs from the nucleus for further cytoplasmic processing during pachytene piRNA biogenesis. However, our data detected neither accumulation of piRNA precursors nor transposon activation, indicating that RANBP9 is dispensable for piRNA biogenesis. Several previous studies have suggested that neither RANBP9 nor RANBP10 is involved in nuclear trafficking because both are lacking the Ran-binding domain, which is critical for binding Ras-like GTPase-RAN [16], [27], [28], [54]. However, our genome-wide RIP-Seq data reveal that many mRNA transcripts bound by RANBP9 are involved in protein/RNA trafficking between the nucleus and the cytoplasm, suggesting that RANBP9 may also function to regulate nucleocytoplasmic transport indirectly by affecting mRNAs encoding nucleocytoplasmic transports. Given that RANBP9 contains multiple functional domains (e.g., PRD, SPRY, LiSH, CTLH, CRA) and can form macromolecular (protein-protein/RNA) complexes with other proteins and RNAs, it is highly likely that RANBP9 acts at multiple levels to regulate the expression of their target and non-target genes. Further functional characterization of each of its multiple domains will help shed more light on the detailed molecular actions of RANBP9.

**Figure 7:**
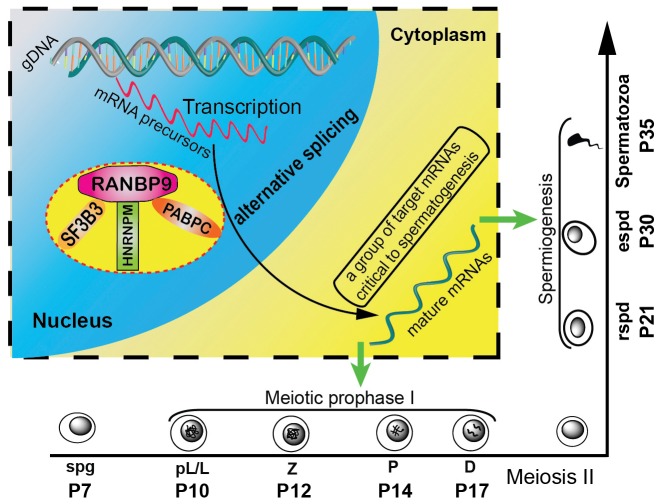
Schematic diagram showing the proposed model of RANBP9 function during spermatogenesis in mice. In the nuclei of spermatocytes (including leptotene, zygotene, pachytene and diplotene) and spermatids, RANBP9 binds key splicing factors (e.g. SF3B3, HNRNPM), and poly(A) binding proteins (PABPC1/2), to coordinate proper alternative splicing of its target mRNA transcripts. Correctly spliced, mature mRNAs are subsequently exported to the cytoplasm to function in spermatocytes and spermatids. Green arrows denote export of processed mRNAs from the nucleus to the cytoplasm.

It has been shown that up to 95% of multi-exon genes generate an average of 3.5 splicing isoforms per gene through alternative splicing [55]. Alternative splicing is particular active in late pachytene spermatocytes and in spermatids, and disruptions in the production of those transcript isoforms are detrimental to successful spermatogenesis [56]. However, the underlying mechanisms remain largely unknown. RANBP9 appears to play a critical role in alternative splicing events during spermatogenesis based on the following lines of evidence reported here: (i) RANBP9 is highly enriched in the nucleus of spermatocytes and spermatids where mRNA precursor processing takes place; (ii) RANBP9 interacts with multiple alternative splicing factors; (iii) altered genome-wide alternative splicing patterns of transcripts (e.g., 2,420 novel transcript isoforms) were detected in the gcKO testes; (iv) ∼22% (154 out of 695) of transcripts with aberrant alternative splicing patterns are bound by, and thus, may be directly targeted by RANBP9. Therefore, we propose a working model in which RANBP9 participates in alternative splicing events in the testis (Figure 7). Briefly, in the nuclei of spermatocytes or spermatids, RANBP9 interacts with key splicing factors (e.g. SF3B3, HNRNPM), and the nuclear PABP proteins (e.g., PABPC1 and PABPC2), to form the protein complexes, which bind >2,300 mRNAs and coordinate their proper splicing and expression. Correctly spliced isoform transcripts are then released to the cytoplasm to fulfill their physiological roles in late meiotic and haploid phases of spermatogenesis.

Although more molecular details need to be delineated in the future, this report, for the first time, defines a novel role of RANBP9 in the post-transcriptional regulation of numerous mRNAs required for normal spermatogenesis, especially spermiogenesis. Moreover, given the severe brain developmental defects in *Ranbp9* global knockout mice, it is expected that conditional inactivation of *Ranbp9* in certain brain cell types would lead to a similar phenotype, and RANBP9 may also play a similar role in brain development.

## Materials and Methods

### Ethics statement

The Institutional Animal Care and Use Committee (IACUC) of the University of Nevada Reno approved the animal use protocol.

### Mouse breeding

B6.FVB-Tg (Stra8-cre)1Reb and 129S.FVB-Tg (Amh-cre)8815Reb/J transgenic mice were purchased from Jackson Laboratory and backcrossed for six generations to C57BL6/J background. Germ cell-specific *Ranbp9* knockout (gcKO) and Sertoli cell-specific *Ranbp9* knockout (scKO) mice were generated according to the strategy as described in Figure 2D. To generate the global *Ranbp9* knockout mice (*Ranbp9^Δ/Δ^*), male *Ranbp9^lox/Δ^* mice derived from *Stra8-Cre;Ranbp9^+/lox^* fathers were crossed with *Ranbp9^lox/Δ^* females to obtain *Ranbp9* ^Δ/Δ^ progeny.

### Histology, TUNEL and western blot assays

Hematoxylin-Eosin (HE) staining, TUNEL staining and Western blot assays were all performed as described [33], [57], [58].

### Immunofluorescence staining

Fresh testis samples were dissected and immediately fixed in 4% paraformaldehyde in 1×PBS solution overnight at 4°C. Then the testis samples were dehydrated by incubation in 10% and 20% sucrose solutions for 2 hrs, respectively followed by embedding into OCT. Cryosections were prepared at 10 µm. For antigen retrieval, slides were boiled in citrate buffer (pH 6.0) for 20 min using a microwave. After a brief wash with 1×PBS, sections were blocked using a solution containing 5% BSA and 5% normal goat serum in 1×PBS at room temperature (RT) for one hour. The first antibodies diluted as appropriate were applied to the sections and incubated overnight at 4°C. Following three 5 min-long washes with 1×PBS at RT, the appropriate secondary antibodies were applied to the sections and incubated for 1 hr at RT. Then the slides were washed with 1×PBS for 3 times again and incubated with a drop of DAPI staining solution (1 ng/µl dissolved in 1×PBS solution) for 5 min at RT. After a wash with 1×PBS for 5 min, the sections were mounted with Aqua-Poly medium (Polysciences, Cat#18606-20) and were ready for confocal microscopic observation and photography.

### Antibodies

The following antibodies were used: Rabbit monoclonal anti-RanBP9 antibody [EPR9920(B)] (ab140627, Abcam), 1∶1000 dilution for immunofluorescence; Mouse monoclonal anti-*Ranbp9* antibody (a kind gift of Dr. Ruggero Pardi)(1∶1000 dilution for western blot; 5 µg for RIP/IP-MS for each sample) [30]; Anti-γH2AX (phospho S139) antibody (ab11174, Abcam) (1∶1000 dilution for immunofluorescence); Anti-ORF1 (LINE1) antibody (a kind gift from Dr. Alex Bortvin) (1∶500 dilution for immunofluorescence); Anti-PABPC1 (a kind gift from Dr. Tadashi Baba)(5 µg was used for each IP reaction) [52]; Anti-PABPC2 (a kind gift from Dr. Tadashi Baba) (7 µg was used for each IP reaction) [52]. The secondary antibodies used for immunofluorescence were as follows: Alexa Fluor 488 Goat Anti-Rabbit IgG, Alexa Fluor 488 Goat Anti-mouse IgG and Alexa Fluor 594 Goat Anti-Rabbit IgG (Invitrogen). HRP-goat anti-mouse IgG (H+L) was purchased from Jackson ImmunoResearch Lab.

### Computer assisted semen assay (CASA)

Sperm were released from cauda epididymides to 1 ml of pre-warmed (37°C) HTF medium by puncturing using a pair of fine forceps, followed by an incubation at 37°C for 30 min. Sperm were then further diluted as appropriate using HTF medium before measurement using a CASA system (Hamilton Throne) as described previously [59].

### Total RNA extraction and quantitative RT-PCR (qPCR)

Total RNA extraction from the whole mouse testes and qPCR setup were performed as described previously [60]. Briefly, 2.5 µg of total RNA from each sample was treated with DNase I to remove residual genomic DNA (DNase-free kit, Invitrogen), and then subject to reverse transcription into first-strand cDNA using a combination of oligo (dT) and random primers. The complement RNA strand was removed by incubation with RNase H enzyme at 37°C for 20 min. For qPCR reaction, 25 ng cDNA was loaded as template in every 20 µl reaction volume for each sample with a 40 amplification cycles. Data were acquired in biological triplicates. Relative gene expression was calculated based on ΔΔCt method using Gapdh as an internal control. All primers sequences were listed in Table S7.

### Co-immunoprecipitation followed by mass spectrometry (IP-MS)

Testes from P30 males were dissected, decapsulated, and lysed in Testis Nuclear Immunoprecipitation buffer (TNIP) containing 20 mM Tris-HCl (pH 7.5), 200 mM NaCl, 2.5 mM MgCl_2_, 0.5% NP-40, 1% Triton X-100, 1 mM DTT, plus freshly supplemented proteinase inhibitor (EDTA-free protease inhibitor tablet, Roche) at an appropriate ratio (100 mg testis per 500 ul TNIP buffer) using an electric tissue homogenizer. The lysate was further subject to 3 rounds of sonication using a Bioruptor 200 (Diagenode) with the following setup: OFF:30 s; ON:30 s; Intensity: L, followed by incubation on ice for 30 min to facilitate complete nuclear lysis. The cellular membrane debris was finally removed by centrifugation at 4°C for 30 min at 20,000 g. For preparation of protein G-coated Dynabeads, 30 ul bed volume of beads for each sample reaction was washed once by incubation with NT2 buffer (20 mM Tris-HCl (pH 7.5), 200 mM NaCl, 2.5 mM MgCl_2_, 0.05% NP-40, 1 mM DTT, plus VRC, an RNase inhibitor purchased from NEB) for 20 min at 4°C, prior to RANBP9 antibody (or IgG control) binding (5 µg antibody per 30 µl beads) through incubating RANBP9 antibody (or IgG) with washed Protein-G-Dynabeads at 4°C for 2 hrs. RANBP9 (or IgG)-coated beads were incubated with pre-cleared testicular lysate at 4°C with overnight gentle shaking. In the next morning, the bead-complex was washed four times with NT2 buffer for 30 min each at 4°C. The protein complex was finally eluted off the beads into 2× Laemmli Sample Buffer and loaded to 4∼12% gradient 1×SDS PAGE gel to visualize all protein bands through the Sypro Ruby staining followed by standard mass spectrometry analysis as described previously [57].

### RNA-Seq

Testes samples collected from 6-week old WT and gcKO mice in biological triplicates were homogenized in Trizol reagent (Life technology) for total RNA extraction as described previously. Prior to sequencing, the total RNA was subject to DNase I treatment (DNase-free, Ambion) to remove trace genomic DNA, followed by the assessment of RNA quality and purity in an Agilent Bioanalyzer 2000 platform. The library preparation and sequencing were completed by the Nevada Genomics Center at the University of Nevada Reno. Starting with 2 µg of total RNA the ribosomal RNA was depleted using Life Technologie's RiboMinus Eukaryote System v2 per manufacturer's instructions. The library preparation was then done using Life Technologie's Ion Total RNA-Seq Kit v2 Library Kit and Ion Xpress RNA-Seq Barcodes, following manufacturer's instructions. Library size and quantitation was established using the Agilent High Sensitivity DNA Kit. Templated ISPs were prepared using Life Technologie's Ion PI Template OT2 200 Kit version 2, following manufacturer's instructions. The sequencing was run on a Life Technologie's Ion Torrent Proton Sequencer using the Life Technologie's Ion PI Sequencing 200 Kit version 2 and Life Technologie's Ion PI v2 Chip, per manufacturer's instructions.

### RNA immunoprecipitation followed by next-generation deep sequencing (RIP-Seq)

For RNA immunoprecipitation, testes of 6-week old mice were dissected and decapsulated in 1×PBS buffer at room temperature. The seminiferous tubules were lysed in a buffer containing 10 mM HEPES (pH 7.0), 100 mM KCl, 5 mM MgCl_2_, 0.5% Triton X-100, 0.5% NP-40, 1 mM DTT, RNaseOUT (100 U/ml) (Invitrogen), VRC (400 µM), plus EDTA-free proteinase inhibitor (Roche) using an electric tissue homogenizer. Testicular lysate was then passed through a 27.5 gauge needle 4 times to promote nuclear lysis, followed by 3 rounds of brief sonication using a Bioruptor 200 (Diagenode) with the following setup: OFF:30 s;ON:30 s; Intensity: L. After incubation on ice for 30 min, the nuclear lysate was pre-cleared by incubation with 50 ul beads at 4°C for 1 hr. For each reaction, 5 µg RANBP9 antibody (or IgG for controls) was incubated with protein G-Dynabeads in 1 ml NT2 buffer (20 mM Tris-HCl (pH 7.5), 200 mM NaCl, 2.5 mM MgCl2, 0.05% NP-40, 1 mM DTT, and VRC) by shaking for 4 hrs at 4°C. Antibody (or control IgG)-coated beads were then incubated with testes nuclear extracts by shaking gently overnight at 4°C. The next morning, the bead complexes containing antibodies, target proteins and RNA were washed for 4 times with 30 min each at 4°C. Protein-bound mRNAs were extracted using RNA Clean & Concentrator-5 kit (ZYMO research) according to the manufacturer's protocol. The integrity and purity of RNA eluted were assessed using an Agilent Bioanalyzer. 1–2 µg of cDNAs were synthesized from the pulled-down RNA using the Clontech SMARTer cDNA kit (Clontech Laboratories, Inc., Mountain View, CA USA, catalog# 634938), and adaptors were removed by digestion with RsaI. Following manufacturer's protocol, the resulting cDNAs were fragmented using an ultrasonicator (Covaris, Inc., Woburn, MA USA), profiled using an Agilent Bioanalyzer, and subjected to Illumina library preparation using NEBNext reagents (New England Biolabs, Ipswich, MA USA, catalog# E6040). The quality, quantity and the size distribution of the Illumina libraries were determined using the Agilent Bioanalyzer. The libraries were then submitted for Illumina HiSeq2000 sequencing (Otogenetics, Norcross, GA). Paired-end 100 nucleotide (nt) reads were generated and checked for data quality using FASTQC (Babraham Institute, Cambridge, UK). The raw data were then subjected to data analysis using Tophat2 and Cufflinks as previously described [61]. Two biological replicates were analyzed for each sample.

### Bioinformatic analyses

RNA-Seq data were processed using Tophat [62] and Cufflinks [63] following a published protocol [64]. Gene ontology (GO) term enrichment analyses were conducted using DAVID [65]. For analyzing alternative splicing patterns based on RNA-Seq data, we developed a pipeline that includes two major steps: first, transcripts with FPKM (fragments per kilobase per million) ≤0.1 were regarded as non-expressed/absent ones, whereas those with FPKM≥1 were defined as expressed ones. Transcripts with FPKM≥1 in gcKO testes and FPKM≤0.1 in WT testes were defined as gcKO-specific/unique transcripts. Transcripts with FPKM≥1 in WT testes and FPKM≤0.1 in gcKO testes were defined as WT-specific/unique transcripts. The standard form of a gene was represented by the transcript with the highest expression in WT testes. Second, sequences of the standard form and the gcKO-specific/unique, homologous isoforms (i.e., partially matching the standard form) were aligned. Insertions were represented by positive length/size values, whereas negative length/size values indicated deletions. The splicing location was determined based on the position within the transcripts rather than their genomic sequences. We used location percentage (splicing location/total transcript size) to reflect the relative position of insertions or deletions along the entire length of the transcripts, e.g. 0% refers to the very 5′end, 50% means the middle of the transcript and 100% indicates the very 3′end.

### Statistics

All data were collected from experiments in biological triplicates and presented as mean ± SEM. Biological significances were determined based on student's *t* test (two groups) or one-way ANOVA unless otherwise stated.

## Supporting Information

Figure S1 *Ranbp9* is dispensable for Sertoli cell development. (A) Gross morphology of the testis and the epididymis of WT and Sertoli cell-specific *Ranbp9* knockout (scKO) mice. (B∼D) Computer-assisted sperm analyses (CASA) on cauda epididymal sperm in WT and scKO mice. Data are presented as mean ± SD, n = 3. No significant differences were found between WT and scKO. (E) Testicular histology of 3-month-old WT and scKO mice. Scale bar = 70 µm. (F) TUNEL staining on WT and scKO testes at the age of 3 months. Apoptotic cells were labeled brown (arrows). Scale bar = 70 µm.
(PDF)

Figure S2 Phase-contrast micrographs showing morphology of the cauda epididymal sperm in WT and gcKO male mice. gcKO mice display teratozoospermia with a wide variety of structural abnormalities in sperm heads, including “head bent back”, “headless flagellum” and aberrantly condensed heads (arrows). Scale bar = 15 µm.
(PDF)

Figure S3 HE staining of paraffin-embedded testicular sections from WT and *Ranbp9 gcKO mice during postnatal development.* The first wave of spermatogenesis appears to be normal in gcKO testes because the morphology and proportions of all types of developing germ cells are comparable between gcKO and WT testes before 6 weeks. However, numerous vacuoles (*) and thinner epithelium (arrowheads) can be readily observed in the testis of 3-month old gcKO mice. Scale bar = 50 µm.
(PDF)

Figure S4 Neonatal lethality and azoospermia in the global *Ranbp9* knockout (*Ranbp9^Δ/Δ^*) mice. (A) Gross morphology of WT and *Ranbp9^Δ/Δ^* littermates at different ages. The body size of *Ranbp9^Δ/Δ^* pups (indicated by red arrows) is noticeably smaller compared to that of the WT littermates. P, postnatal day. (B) Gross morphology of WT and *Ranbp9^Δ/Δ^* testes and epididymides at P60. Scale bar = 0.5 cm. (C) Comparison of body weight between WT and *Ranbp9^Δ/Δ^* mice at P60. Data are presented as mean ± SD, n = 3. (D) Comparison of testis weight between WT and *Ranbp9^Δ/Δ^* mice at P60. Data are presented as mean ± SD, n = 3. (E) The testis/body weight index defined as the ratio of testis weight (mg) vs. body weight (g). Data are presented as mean ± SD, n = 3. (F) The growth curve of body weight during postnatal development between WT and *Ranbp9^Δ/Δ^* mice. Data are presented as mean ± SD, n = 3. (G) HE staining of paraffin-embedded testicular sections of WT and *Ranbp9^Δ/^* ^Δ^ mice at P60. Scale bar = 40 µm. (H) HE staining of paraffin-embedded cauda epididymal sections of WT and *Ranbp9^Δ/Δ^* mice at P60. Scale bar = 40 µm.
(PDF)

Figure S5 *Ranbp9* is not involved in piRNAs-mediated transposon repression. (A) Immunohistochemical staining of LINE1 ORF1 in gcKO and *Miwi2* knockout testes (serving as a positive control). ORF1 is not detectable in gcKO testes while it is highly expressed in *Miwi2* knockout testes, which display transposon de-suppression. Scale bar = 60 µm. (B∼C) qPCR analyses on levels of DNA transposons and retrotransposons in testis and liver (serving as a somatic tissue control) among WT, gcKO and *Ranbp9^Δ/Δ^* mice at P30. Data are presented as mean ± SD, n = 3. (D) RT-PCR detection of four piRNAs precursors in WT and gcKO testes. *Gapdh* serves as a loading control. NTC, non-template control.
(PDF)

Table S1 Multi-alignment analyses of orthologous RANBP9 in 10 eukaryotic species.
(PDF)

Table S2 Significantly de-regulated genes expressed in the gcKO testes compared to the WT controls (cutoff: P<0.05, fold change>2).
(XLS)

Table S3 A summary of genes with various splicing events (deletions/insertions at the gene body, the 3′ UTR or the 5′UTR).
(XLSX)

Table S4 List of transcript isoforms unique to gcKO testes.
(XLSX)

Table S5 RANBP9-bound target mRNA transcripts identified by RIP-Seq.
(XLS)

Table S6 Fold changes of RANBP9-bound target mRNAs in gcKO testes based on RNA-Seq.
(XLS)

Table S7 Sequences of primers used in this study.
(XLSX)
