# Supplementary figures and images for: RAN-Binding Protein 9 is Involved in Alternative Splicing and is Critical for Male Germ Cell Development and Male Fertility
Source: PLoS Genet. 2014 Dec 4;10(12):e1004825. doi: 10.1371/journal.pgen.1004825 (PMC4256260; doi:10.1371/journal.pgen.1004825)

Figure S1

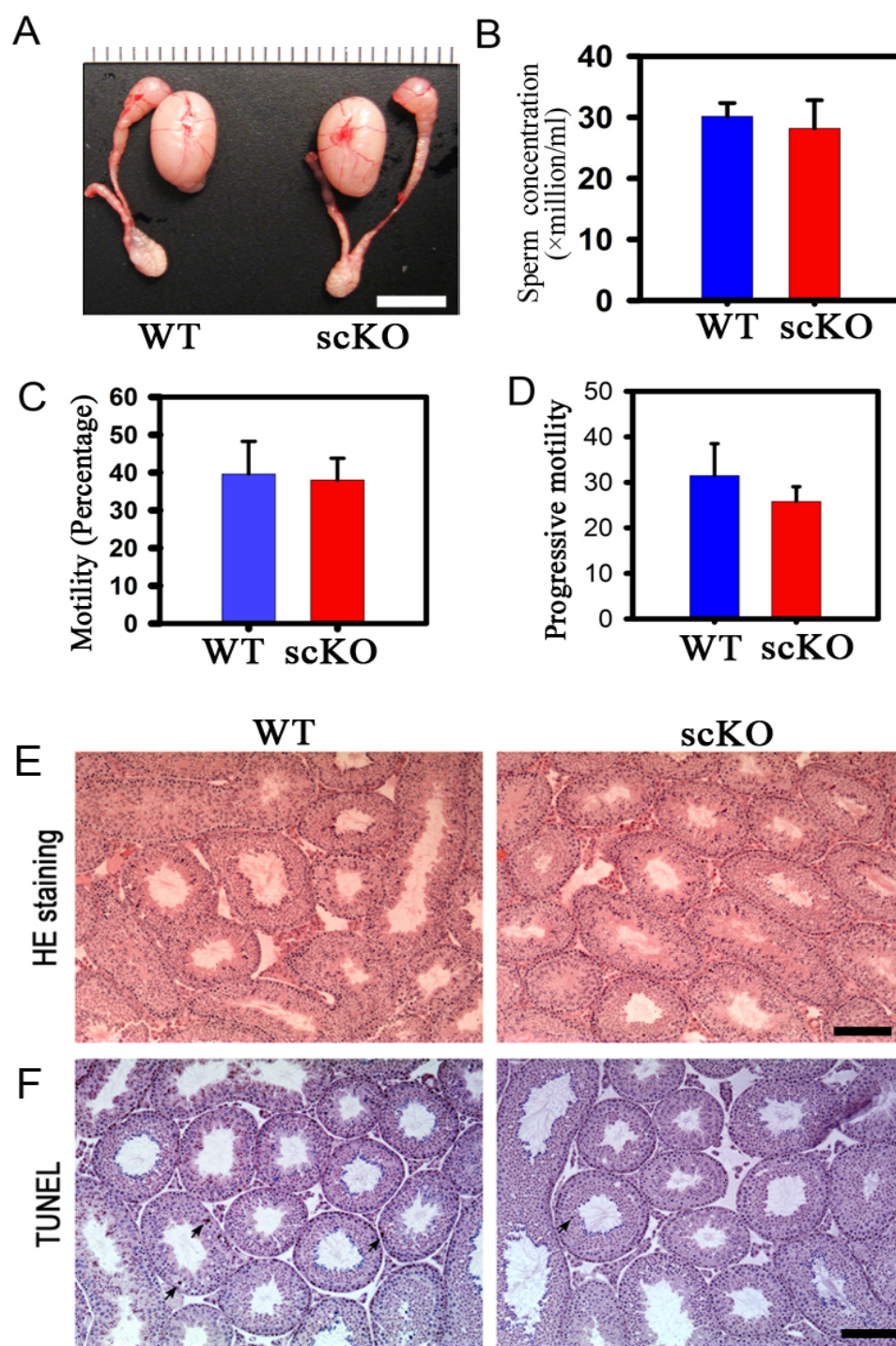

Supplement: Figure S1 — Ranbp9 is dispensable for Sertoli cell development. (A) Gross morphology of the testis and the epididymis of WT and Sertoli cell-specific Ranbp9 knockout (scKO) mice. (B∼D) Computer-assisted sperm analyses (CASA) on cauda epididymal sperm in WT and scKO mice. Data are presented as mean ± SD, n = 3. No significant differences were found between WT and scKO. (E) Testicular histology of 3-month-old WT and scKO mice. Scale bar = 70 µm. (F) TUNEL staining on WT and scKO testes at the age of 3 months. Apoptotic cells were labeled brown (arrows). Scale bar = 70 µm. (PDF) [file pgen.1004825.s001.pdf]

Figure S2

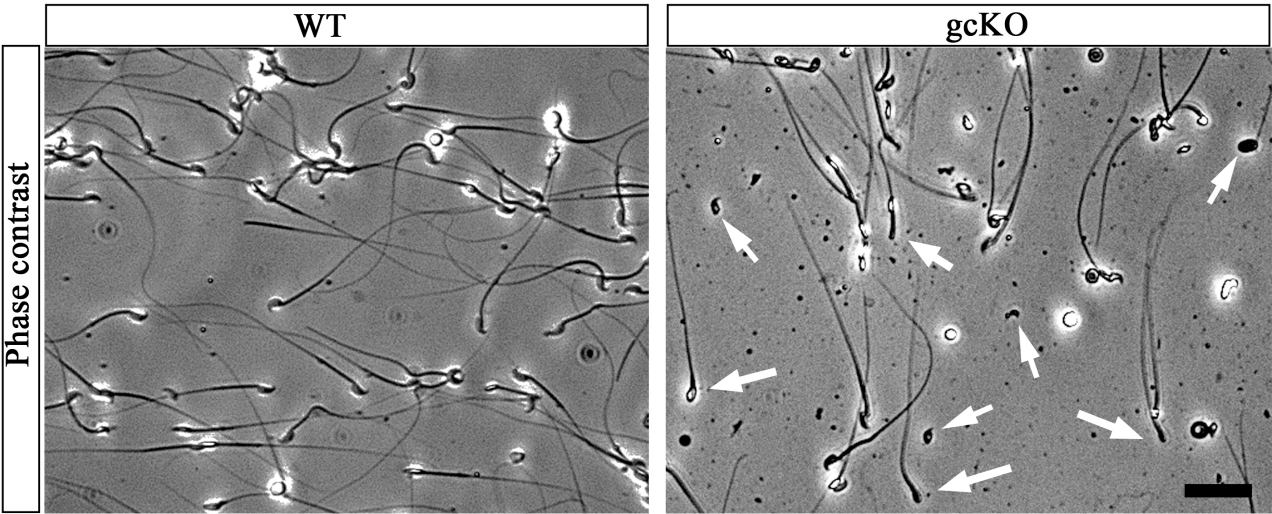

Supplement: Figure S2 — Phase-contrast micrographs showing morphology of the cauda epididymal sperm in WT and gcKO male mice. gcKO mice display teratozoospermia with a wide variety of structural abnormalities in sperm heads, including “head bent back”, “headless flagellum” and aberrantly condensed heads (arrows). Scale bar = 15 µm. (PDF) [file pgen.1004825.s002.pdf]

Figure S3

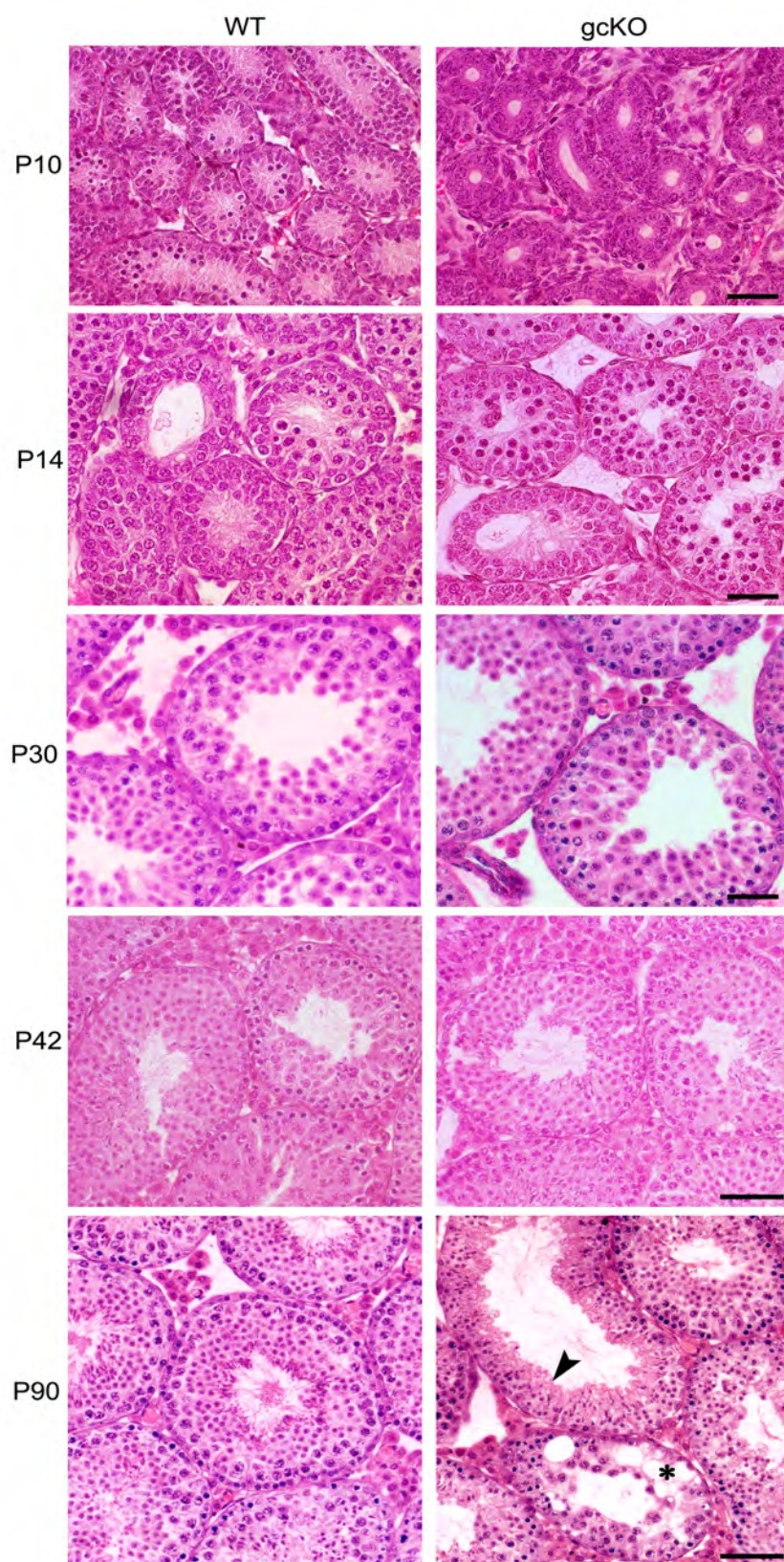

Supplement: Figure S3 — HE staining of paraffin-embedded testicular sections from WT and Ranbp9 gcKO mice during postnatal development. The first wave of spermatogenesis appears to be normal in gcKO testes because the morphology and proportions of all types of developing germ cells are comparable between gcKO and WT testes before 6 weeks. However, numerous vacuoles (*) and thinner epithelium (arrowheads) can be readily observed in the testis of 3-month old gcKO mice. Scale bar = 50 µm. (PDF) [file pgen.1004825.s003.pdf]

Figure S4

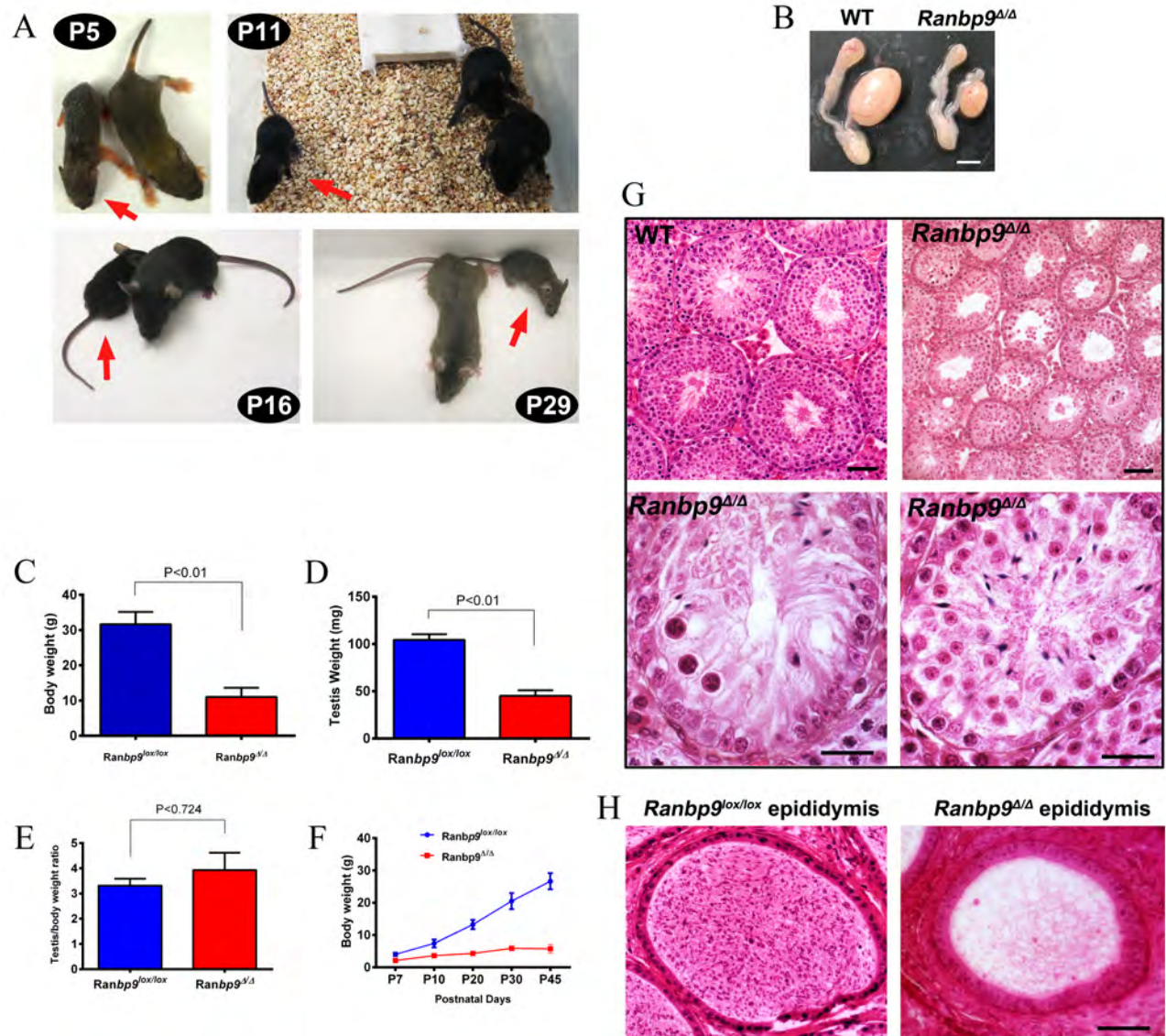

Supplement: Figure S4 — Neonatal lethality and azoospermia in the global Ranbp9 knockout (Ranbp9Δ/Δ) mice. (A) Gross morphology of WT and Ranbp9Δ/Δ littermates at different ages. The body size of Ranbp9Δ/Δ pups (indicated by red arrows) is noticeably smaller compared to that of the WT littermates. P, postnatal day. (B) Gross morphology of WT and Ranbp9Δ/Δ testes and epididymides at P60. Scale bar = 0.5 cm. (C) Comparison of body weight between WT and Ranbp9Δ/Δ mice at P60. Data are presented as mean ± SD, n = 3. (D) Comparison of testis weight between WT and Ranbp9Δ/Δ mice at P60. Data are presented as mean ± SD, n = 3. (E) The testis/body weight index defined as the ratio of testis weight (mg) vs. body weight (g). Data are presented as mean ± SD, n = 3. (F) The growth curve of body weight during postnatal development between WT and Ranbp9Δ/Δ mice. Data are presented as mean ± SD, n = 3. (G) HE staining of paraffin-embedded testicular sections of WT and Ranbp9Δ/ Δ mice at P60. Scale bar = 40 µm. (H) HE staining of paraffin-embedded cauda epididymal sections of WT and Ranbp9Δ/Δ mice at P60. Scale bar = 40 µm. (PDF) [file pgen.1004825.s004.pdf]

Figure S5

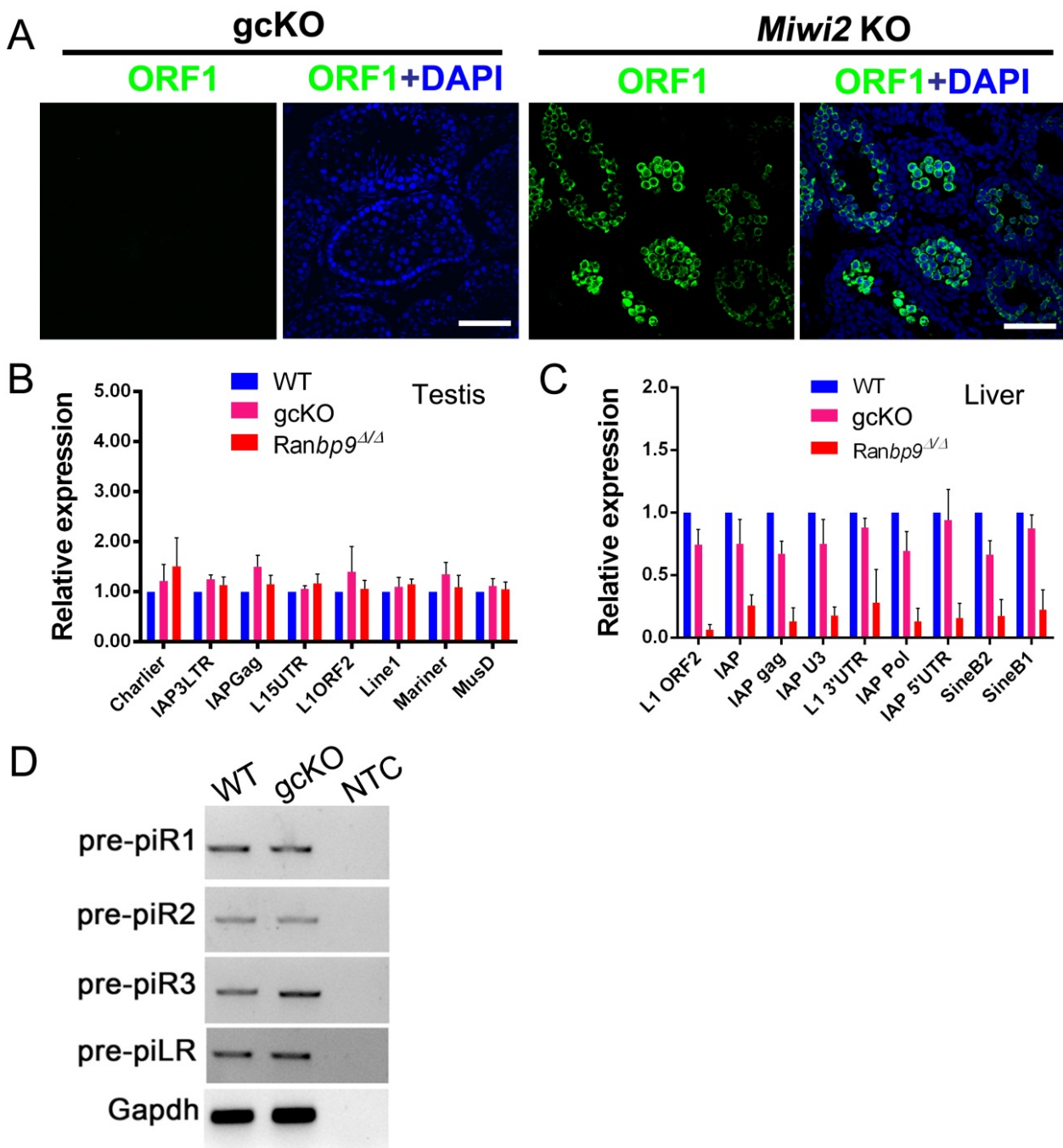

Supplement: Figure S5 — Ranbp9 is not involved in piRNAs-mediated transposon repression. (A) Immunohistochemical staining of LINE1 ORF1 in gcKO and Miwi2 knockout testes (serving as a positive control). ORF1 is not detectable in gcKO testes while it is highly expressed in Miwi2 knockout testes, which display transposon de-suppression. Scale bar = 60 µm. (B∼C) qPCR analyses on levels of DNA transposons and retrotransposons in testis and liver (serving as a somatic tissue control) among WT, gcKO and Ranbp9Δ/Δ mice at P30. Data are presented as mean ± SD, n = 3. (D) RT-PCR detection of four piRNAs precursors in WT and gcKO testes. Gapdh serves as a loading control. NTC, non-template control. (PDF) [file pgen.1004825.s005.pdf]
