# Supplementary material for: RAN-Binding Protein 9 is Involved in Alternative Splicing and is Critical for Male Germ Cell Development and Male Fertility
Source: PLoS Genet. 2014 Dec 4;10(12):e1004825. doi: 10.1371/journal.pgen.1004825 (PMC4256260; doi:10.1371/journal.pgen.1004825)
Supplement: Table S1 — Multi-alignment analyses of orthologous RANBP9 in 10 eukaryotic species. (PDF) [file pgen.1004825.s006.pdf]

Table S1

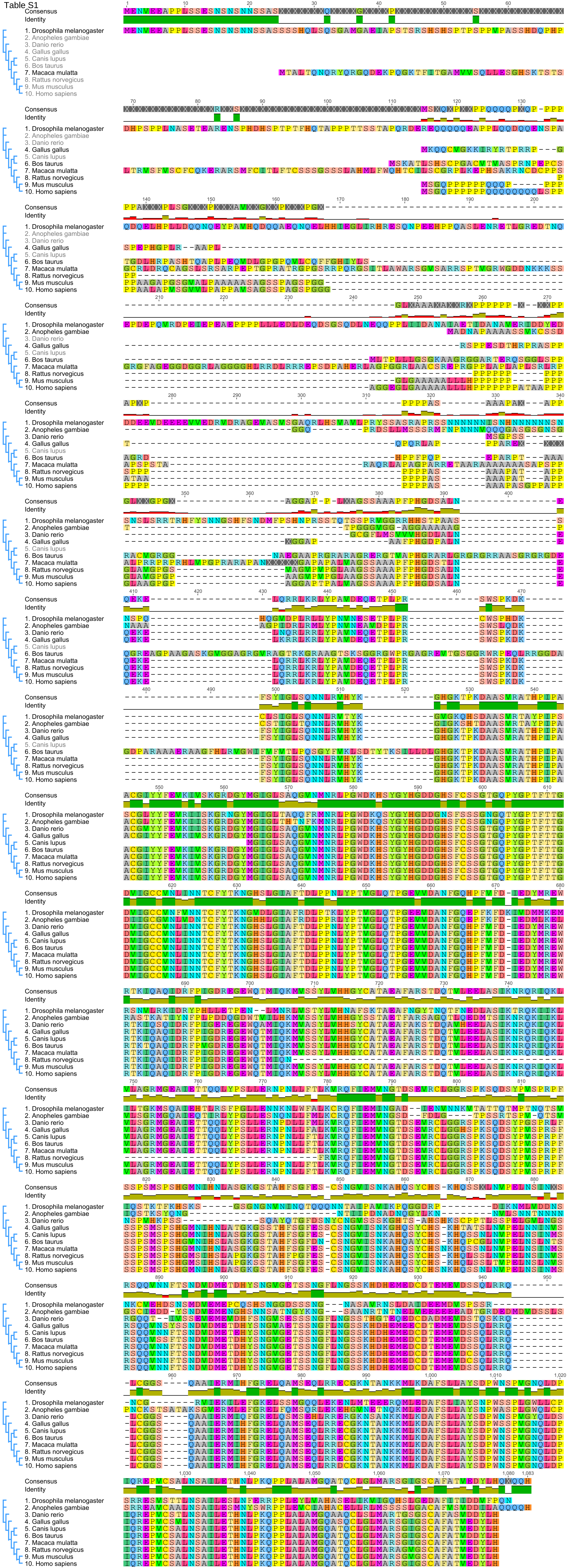
Consensus
Identity
1. Drosophila melanogaster
2. Anopheles gambiae
3. Danio rerio
4. Gallus gallus
5. Canis lupus
6. Bos taurus
7. Macaca mulatta
8. Rattus norvegicus
9. Mus musculus
10. Homo sapiens
